# Marfan Syndrome: Enhanced Diagnostic Tools and Follow-up Management Strategies

**DOI:** 10.3390/diagnostics13132284

**Published:** 2023-07-05

**Authors:** Susan Marelli, Emanuele Micaglio, Jacopo Taurino, Paolo Salvi, Erica Rurali, Gianluca L. Perrucci, Claudia Dolci, Nathasha Samali Udugampolage, Rosario Caruso, Davide Gentilini, Giuliana Trifiro’, Edward Callus, Alessandro Frigiola, Carlo De Vincentiis, Carlo Pappone, Gianfranco Parati, Alessandro Pini

**Affiliations:** 1 Cardiovascular-Genetic Center, IRCCS Policlinico San Donato, 20097 Milan, Italyalessandro.pini@grupposandonato.it (A.P.); 2 Arrhythmia and Electrophysiology Department, IRCCS Policlinico San Donato, 20097 Milan, Italy; emanuele.micaglio@grupposandonato.it (E.M.); carlo.pappone@grupposandonato.it (C.P.); 3 Istituto Auxologico Italiano, Cardiology Unit, IRCCS, 20133 Milan, Italy; psalvi@gmail.com (P.S.);; 4 Unit of Vascular Biology and Regenerative Medicine, Centro Cardiologico Monzino IRCCS, 20138 Milan, Italy; erica.rurali@cardiologicomonzino.it (E.R.);; 5 Laboratory of Functional Anatomy of the Stomatognathic System (LAFAS), Department of Biomedical Sciences for Health, Università degli Studi di Milano, 20133 Milan, Italy; claudia.dolci@unimi.it; 6 Clinical Research Service, IRCCS Policlinico San Donato, 20097 Milan, Italy; rosario.caruso@grupposandonato.it; 7 Department of Biomedical Sciences for Health, University of Milan, 20133 Milan, Italy; edward.callus@unimi.it; 8 Department of Brain and Behavioral Sciences, University of Pavia, 27100 Pavia, Italy; davide.gentilini@unipv.it; 9 Bioinformatics and Statistical Genomics Unit, Istituto Auxologico Italiano IRCCS, Cusano Milanino, University of Milano-Bicocca, 20095 Milan, Italy; 10 Clinical Psychology Service, IRCCS Policlinico San Donato, 20097 Milan, Italy; 11 Department of Congenital Cardiac Surgery, IRCCS Policlinico San Donato, San Donato Milanese, 20097 Milan, Italy; alessandro.frigiola@grupposandonato.it; 12 Association “Bambini Cardiopatici nel Mondo” Non-Governmental Organization (NGO), 20123 Milan, Italy; 13 Department of Cardiothoracic, Vascular Anaesthesia and Intensive Care, IRCCS Policlinico San Donato, 20097 Milan, Italy; 14 Department of Cardiac Surgery, IRCCS Policlinico San Donato, 20097 Milan, Italy; 15 Institute of Molecular and Translational Cardiology, IRCCS Policlinico San Donato, 20097 Milan, Italy; 16 Faculty of Medicine and Surgery, Vita-Salute San Raffaele University, 20132 Milan, Italy; 17 Department of Medicine and Surgery, University of Milano-Bicocca, 20126 Milan, Italy

**Keywords:** Marfan syndrome, connective tissue disease, multidisciplinary approach, personalized medicine, Fibrillin-1

## Abstract

Marfan syndrome (MFS) is a rare inherited autosomic disorder, which encompasses a variety of systemic manifestations caused by mutations in the Fibrillin-1 encoding gene (*FBN1*). Cardinal clinical phenotypes of MFS are highly variable in terms of severity, and commonly involve cardiovascular, ocular, and musculoskeletal systems with a wide range of manifestations, such as ascending aorta aneurysms and dissection, mitral valve prolapse, ectopia lentis and long bone overgrowth, respectively. Of note, an accurate and prompt diagnosis is pivotal in order to provide the best treatment to the patients as early as possible. To date, the diagnosis of the syndrome has relied upon a systemic score calculation as well as DNA mutation identification. The aim of this review is to summarize the latest MFS evidence regarding the definition, differences and similarities with other connective tissue pathologies with severe systemic phenotypes (e.g., Autosomal dominant Weill–Marchesani syndrome, Loeys–Dietz syndrome, Ehlers–Danlos syndrome) and clinical assessment. In this regard, the management of MFS requires a multidisciplinary team in order to accurately control the evolution of the most severe and potentially life-threatening complications. Based on recent findings in the literature and our clinical experience, we propose a multidisciplinary approach involving specialists in different clinical fields (i.e., cardiologists, surgeons, ophthalmologists, orthopedics, pneumologists, neurologists, endocrinologists, geneticists, and psychologists) to comprehensively characterize, treat, and manage MFS patients with a personalized medicine approach.

## 1. Introduction

Marfan syndrome (MFS, OMIM #154700) is a common genetic disorder that affects mainly connective tissues, due to heterozygous mutations in *FBN1*, a relatively large gene (230 kb long) composed of 65 exons located on the long arm of chromosome 15 (15q21.1) that encodes for Fibrillin-1, a protein component of the Extracellular Matrix (ECM) [1]. The estimated prevalence of MFS is 1:3000–1:5000, without differences in prevalence between sex or ethnic groups. MFS comprises a broad phenotypic continuum ranging from mild (features of Marfan syndrome in one or a few systems) to severe and rapidly progressive neonatal multi-organ disease [2].

Cardinal clinical phenotypes (characteristic but highly variable) linked to the disorder involve the cardiovascular, ocular, and musculoskeletal systems. It was first reported in 1896, when Antoine-Bernard Marfan described the syndrome in the Victor McKusick monograph. In the following years, more and more features were added until a draft of a list of mandatory diagnostic criteria was established. The timing at which clinical symptoms appear can vary significantly in individuals, ranging from severe cardiovascular complications present at birth in the neonatal form, to patients developing manifestations later in life. If left untreated, the average lifespan for patients is typically around 40 years [3]. However, the past 30 years have brought about considerable advancements in management techniques and research, leading to a significant increase in patients’ life expectancy. The factors that have been reported to expedite the progression of aortic dilatation or dissection include elevated blood pressure, strenuous physical exertion (especially isometric sports activity), and pregnancy.

### 1.1. Clinical Diagnosis

The early diagnosis of MFS allows for the initiation of proactive treatment approaches involving beta-blockers and angiotensin I (AT-1) antagonists, which can effectively halt the progression of aortic root dilation. This not only diminishes the requirement for surgery, but also mitigates potential life-threatening scenarios. To date, despite the existence of a targeted genetic test, the diagnosis of MFS is based on specific clinical criteria; the Ghent criteria were codified in 1996 and later revised in 2010 [4]. According to the 1996 Ghent criteria, the diagnosis requires at least the presence of a major criterion in two different systems (aortic root dilatation/aneurysm, ectopia lentis, dural ectasia, severe skeletal involvement) and one minor criterion in a third system (myopia, mitral valve prolapse, mild skeletal involvement, skin striae, and pneumothorax). The revised Ghent criteria set out in 2010 give more weight to aortic root involvement and ectopia lentis as cardinal features, leading to the definitive diagnosis of MFS when these symptoms manifest together. Skeletal features, myopia, skin striae, mitral valve prolapse, spontaneous pneumothorax, and dural ectasia are grouped together in the “systemic score”. A score is assigned to each item: if the sum of scores reaches seven or more, the system score becomes relevant for the diagnosis. The most relevant difference between the 1996 and 2010 Ghent Criteria is the shift of dural ectasia from a “major” to a “minor” criterion. Moreover, when evaluating skeletal features, a lot of importance is given to the combined wrist and thumb sign, acetabular protrusion, hindfoot deformity, and pectus carinatum. Positive family history, as well as pathogenic *FBN1* mutation, are important for a definitive diagnosis.

According to the 2010 Ghent revisited criteria, the diagnosis of MFS is possible in the presence of (A) aortic root dilatation/dissection + ectopia lentis; (B) aortic root dilatation/dissection + *FBN1* mutation; (C) ectopia lentis + *FBN1* mutation (known to have been previously associated with aortic root dilatation in the literature, or present in the family) [4].

Due to age-dependent manifestations and the difficulty of making a diagnosis in pediatric patients with a negative family history [5], the Kid Short Marfan score (Kid-SMS) was developed. It is an easily executable tool for the risk stratification of pediatric patients suspected of MFS. Children are classified into three categories based on risk: very high risk, high risk, and moderate risk. When the diagnosis cannot be confirmed, Kid-SMS recommends a safe follow-up regime. The following manifestations require consideration: aortic root dilatation, ectopia lentis, mitral valve prolapse, tricuspid valve prolapse, pulmonary artery dilatation, and skeletal features [5]. Although Kid-SMS also predicts a risk of MFS in some patients without disease, it can potentially identify patients with other syndromes, such as Loeys–Dietz or Ehlers–Danlos syndromes, which could be confused with MFS and also require regular follow-up [6].

MFS is an inherently complex condition, with significant inter- and intrafamilial variability. The disorder manifests as a spectrum of diverse symptoms across various organs, differing greatly from the classical patient profile. Several of the physical findings associated with MFS can also be seen in the general population [7] or in other syndromic Heritable Thoracic Aortic Diseases (H-TAD), such as Loeys–Dietz syndrome (LDS) and Ehlers–Danlos syndrome (EDS). This clinical heterogeneity, combined with manifestations appearing in childhood, the prevalence of *FBN1* mutations across various fibrillinopathies, and a high occurrence of de novo mutations, can pose diagnostic challenges. Some patients may require longer observation periods before reaching a definitive diagnosis, which underscores the importance of an integrated, multidisciplinary approach—drawing on dysmorphology, cardiology, ophthalmology, and radiology—for the accurate identification of the condition. The main features detected in MFS are listed below.

Cardiovascular features. The primary causes of morbidity in MFS stem from cardiovascular complications: aortic dilation at the sinuses of Valsalva, susceptibility to aortic tears and ruptures, mitral and tricuspid valve prolapse, and enlargement of the proximal pulmonary artery [2]. Aortic aneurysm is one of the pivotal features in the clinical diagnosis of Marfan syndrome, and the dissection of such an aneurysm poses the greatest threat to life.

The sinus of Valsalva is found to be dilated in approximately 80% of adult MFS patients, creating a characteristic pear-shaped aortic root. The onset and rate of this aortic dilation are highly unpredictable, ranging from rare prenatal onset to cases where critical dimensions are never reached. A comparison with age-dependent benchmarks is necessary to determine if aortic measurements, especially in pediatric patients, fall within the normal range. However, it is generally accepted that aortic roots measuring ≥ 40 mm in diameter in adults signify dilation. Studies report that in the MFS population, without preventive surgery, the most prevalent type of aortic dissection is type A, which involves the aortic root and frequently the descending aorta [8]. The risk of dissection depends on the aortic diameter (the bigger the diameter is, the more relevant the risk becomes); few cases of dissections are reported in patients with mild aortic dilatation or even with no dilatation. In addition, the rate of aortic growth and family history are included among risk factors. MFS patients with surgical aortic root replacement commonly face the onset of aneurysms and/or dissections along the arterial tree [9].

In MFS, aortic dilatation undergoes time-related progression. Histological evaluations of the aortic media have revealed the fragmentation of elastic fibers, a decrease in elastin content, and an accumulation of shapeless matrix components. Although this disease can cause aortic aneurysms, “cystic medial necrosis” does not allow for a distinction to be made from other causes. A high risk of aortic dissection or rupture is reached when the maximal dimension of the aortic root is nearly 5.0 cm. There is extreme variability in the origin and trend of growth of aortic dilatation. Secondary aortic regurgitation is caused by a stretched aortic annulus due to the enlargement of the aneurysm. Rare cases of aortic dissection have been reported in childhood [10].

Other cardiovascular manifestations include aortic valvular dysfunction that can cause secondary left ventricular dilatation and failure caused by volume overload. Additionally, severe pediatric MFS patients are enlisted for cardiovascular surgery if there is mitral valve prolapse with congestive heart failure; indeed, it is also one of the leading causes of cardiovascular morbidity and mortality in this population [10].

Moreover, prevalence rates of 14% for intracranial aneurysm and 3% for intracranial dissection were found in MFS [11].

Ocular features—Ectopia lentis is one of the cardinal characteristics of MFS, found in about 60% of patients. It is due to the weakness of the ciliary zonulae (the suspensory ligament of the lens). Myopia is the most common ocular minor feature, and often progresses rapidly during childhood: it is found in 34–44% of MFS patients compared to the general population [12]. Other clinical ocular manifestations may occur through increased axial globe length and corneal flatness [13]. Studies have shown findings implying strabismus, exotropia, esotropia, vertical deviations, and primary inferior oblique muscle over action and glaucoma [14].

Skeletal, bone, and muscle features—the facial gestalt includes a long and narrow face with deeply set eyes, malar hypoplasia, abnormal ear cartilage, down slanting of the palpebral fissures, micro/retrognathia, high arch narrow palate, and tooth crowding.

The prevalence of typical facial features in MFS is not clearly defined, with large differences among studies, but these features are reported in up to 83% of young patients with MFS [15]. Recent studies have highlighted the importance of early recognition of the facial features in MFS (i.e., dolichocephalic head-forms), especially in the infantile population, in order to provide effective management and follow-up of the disease [15].

In MFS, the skeletal system is typically characterized by joint laxity and abnormal linear growth patterns. This results in a noticeable disproportion, known as dolichostenomelia, between limb length and trunk size, leading to an enlarged arm span-to-height ratio and a diminished upper-to-lower segment ratio While individuals with Marfan syndrome may not necessarily stand out as tall according to general population standards, they often exhibit a height exceeding what would be anticipated based on their familial height trends [15].

Abnormal rib growth in Marfan syndrome can result in conditions such as pectus excavatum or pectus carinatum. Over 63% of MFS patients exhibit scoliosis exceeding 10°, often accompanied by thoracic lordosis, lumbar kyphosis, severe spondylolisthesis, dural ectasia, and pedicle dystrophy [16]. During growth spurts, scoliosis may evolve rapidly, leading to significant deformity [17]. The syndrome’s characteristic bone overgrowth and joint laxity manifest in distinctive thumb and wrist signs, a condition known as arachnodactyly. The presence of flat feet (pes planus) is a consequence of the inward rotation of the medial aspect of the ankle, and an excessive arch to the foot (pes cavus) can be found as well. Moreover, some patients can show reduced joint mobility, in particular of the elbow and fingers. An abnormal depth of the acetabulum (protrusio acetabuli) is diagnosed in 31% of individuals with MFS.

Decreased bone mineral density has been reported in adults [18] and pediatric patients. In fact, a study [19] demonstrated that young patients with MFS have lower than average bone mineral density at the lumbar spine and femur; it tends to decrease over time. In the same way, muscle mass measured by DXA is decreased in children with MFS and worsens from childhood to adulthood compared to healthy age-matched controls [20]. According to another study [21], the incidence of fractures is higher in children patients with MFS because of the low mineral density compared to the general population of the same age and location.

Dura—dural ectasia is the straining of the dural sac in the lumbosacral region, causing bone erosion and nerve sequestration [22]. It causes lower backache, which usually radiates to the proximal leg, resulting in fatigue, numbness of the distal legs, and pain in the genital or rectal regions. A leak in the cerebrospinal fluid (CSF) from the dural sac can cause a postural drop in CSF pressure, leading to headache; in some patients, CFS hypotension can be so severe that it requires hospitalization [23].

Skin features. Skin stretch marks, umbilical hernia, inguinal hernia (often congenital), and rectus abdominal muscle diastasis are frequent. Individuals may exhibit an insufficiency of muscularity and fat stores despite adequate caloric intake.

The lung and respiratory system features—the lung manifestations of MFS can lead to substantial disability and reduced quality of life. Chest deformity and respiratory and muscle weakness contribute to restrictive lung disease. Reduced aerobic capacity, increased total and residual lung volume, and reduced peak oxygen uptake can be found as respiratory difficulties [24]. Parenchymal lung disease leads to upper lobe blebs and can predispose one to spontaneous pneumothorax, especially during adolescence and young adult age. Both chest wall deformities and airway wall defects can manifest disorders such as asthma and bronchiectasis. Finally, sleep-disordered breathing can manifest in the presence of laxity of the soft tissue, along with a soft tissue laxity and a predisposition for upper airway obstruction [25].

Psychological features—notwithstanding scientific progresses in the genetic field, there is still a lack of a proper definition for the type of influence that genetic diseases may have in the life experiences of these patients [26], although there are strong indications that patients with genetic disorders need support in the process of adaptation and existential re-organization subsequent to the diagnosis [27].

To date, psychological studies have highlighted a decrease in the existential level of satisfaction among the MFS population [28], with a relevant impact on the quality of life (QoL) and social relationships, and an association of the condition with a negative perception of the health status, including both the physical and psychological spheres. Moreover, patients with MFS are more likely to have significant levels of stress and anxiety [29] and to suffer from depression [30]. Other studies have shown a potential correlation between chronic pain and physical weakness among MFS patients [31]. Additionally, coping strategies have been found in studies that included patients with genetic-related aortic diseases [32].

The subjective perception of the disease is not always associated with the actual clinical–pathological expression of the disease [33]; as a consequence, it is important to identify psychological features and coping mechanisms linked to disease awareness and management.

Considering the variability of the MFS clinical expression and the impact the disease could have on different aspects of a person’s life, psychological or psychotherapeutic support plays a pivotal role. A multidisciplinary approach that considers the psychological implications of the disease when taking care of MFS patients is highly advised [29,34]. Psychological assistance is more relevant if targeted to those who have experienced cardiovascular interventions [29,35].

### 1.2. Genetic and Molecular Relevance

#### 1.2.1. The Genetic Basis of MFS

MFS is inherited in an autosomal dominant way; this means that affected individuals have a 50% chance of transmitting the causing mutation (and thus the disease) to their children, regardless of gender. It has been estimated that approximately 75% of MFS patients inherit the disease from an affected parent, while the remaining 25% obtain the disorder due to a de novo pathogenic variant. Nonetheless, it has been shown that 0.5% of MFS patients carry homozygous or compound heterozygous mutations in *FBN1* [36].

Pathogenic variants in *FBN1* are expected in about 82–83% of patients who meet the 2010 Ghent revised criteria for MFS [37,38].

The relative frequency of the reported *FBN1* variants is about 66% for missense variants, 10–15% for small insertions, deletions, or duplications, and 10–15% for splicing errors most commonly affecting canonical splice sequences at exon/intron boundaries [39]. Larger rearrangements, including both deletions and insertions, although a minority, have been found in 3–7% of patients [40,41,42], while entire gene deletions are much rarer [43].

These mutations can be classified into two groups: the first one includes *FBN1* haploinsufficiency mutations (HI), which represent about one-third of the *FBN1* mutations (this category includes nonsense mutations, splice site mutations, or small duplications/deletions leading to out-of-frame events too). HI are presumed to cause a diminished amount of available Fibrillin-1 protein in the extracellular matrix. The second group includes the other two-thirds of possible mutations, which are dominant and negative (DN), namely, missense mutations, splice site mutations, or small duplications/deletions leading to in-frame reading events. DN mutations lead to the formation of abnormal Fibrillin-1, which prevents the normal function of the extracellular matrix [44]. However, the effect of a specific mutation can only be predicted [44].

*FBN1* mutations have high and age-dependent penetrance; to date, no pedigree document shows any precedents of non-penetrance. Yet, related individuals carrying an identical *FBN1* mutation vary widely with respect to age at onset, organ system involvement, and disease severity.

Moreover, some mutations have also been associated with different phenotypes, such as non-syndromic TAAD [45] and others (see below) [46].

Although there are numerous previously published studies on *FBN1* genotyping, solid conclusions about the genotype–phenotype correlation have not yet been reached, and so it is not possible to predict the severity of manifestations using only the mutation identified in MFS patients, with a few exceptions: mutations within the middle region of the gene (exon 24–32) seem to correlate with an earlier onset and a more severe phenotype [47], while variants that alter cysteine residues are instead associated with a high probability of aortic dilation/dissection, mitral valve prolapse, or Ectopia lentis [48]. Aortic events in the youngest population are highly related to truncating and splice-altering mutations [37], while patients with a stop codon variant have more frequent skeletal and skin involvement [49].

It is possible that the high phenotypic variability observed in MFS, even among individuals of the same families, may be due to other genetic or epigenetic modifiers. In this regard, a recent study by Gentilini et al. [50] showed that both rare and common variants in the *FBN1* gene and in the other 11 genes that play a role in connective tissue diseases have an effect that significantly contributes to the complexity of the clinical spectrum in MFS patients. Furthermore, a notable correlation has been observed between the number of clinical symptoms in Marfan syndrome patients and the number of identified rare variants.

#### 1.2.2. The Molecular Basis of MFS

The ECM is commonly defined as a dynamic network of interconnected supramolecular aggregates such as elastic fibers, proteoglycan aggregates, collagen fibrils, and fibrillin micro fibrils. Each macromolecule supports specific structural and regulatory functions in the ECM [51]. Elastic fibers, found in various tissues such as the skin, lungs, arteries, and ligaments, are vital for providing elasticity and resilience [52]. fibrillin microfibrils, a critical component of these elastic fibers, interact with various cellular and ECM components, including integrins, bone morphogenetic proteins (BMPs), and the large latent complex of the transforming growth factor-β (TGFβ) [53]. The biological role performed by fibrillin microfibrils is tissue-dependent and aims not only at tissue architecture organization and repair, but also at sequestering a variety of growth factors. Thus, fibrillin microfibrils are primary players as both ECM structural and biochemical regulators in physiological and pathological conditions [46,54].

In more detail, Fibrillin-1, encoded by the *FBN1* gene [55], is a large glycoprotein constituted by 7 TGF-β binding protein-like domains and 47 epidermal growth factor (EGF)-like domains [56], playing a crucial role in microfibril stability and assembly [57,58]. Since TGF-β cytokines are secreted in Large Latent Complexes (LLC) that contain Latency-Associated Peptide (LAP) and latent TGF-β binding protein (LTBP) anchored by fibrillin-1 to the ECM, Fibrillin-1 can orchestrate TGF-β activation [59]. In physiological conditions, inflammatory proteolytic enzymes and the determined physiological stimuli can lead to microfibril degradation, which is responsible for local TGF-β activation. On the contrary, mutated Fibrillin-1 in MFS causes the fragmentation of microfibrils responsible for massive TGF-β release and the subsequent overactivation of its downstream signaling cascades, which represents the core of MFS pathogenesis [60].

TGF-β specifically stimulates collagen production, tightly manages ECM remodeling, and triggers tissue fibrosis, which jeopardizes the structure and function of organs [61]. The fragmentation and disorganization of elastic fibers, fibrosis with collagen production, accumulation of amorphus matrix components, and loss of cell nuclei [62] collectively contribute to the onset of medial cystic necrosis. This is a feature often seen in the medial layer of the aortic wall in MFS patients. In the frame of studies on new biological targets potentially involved in the detrimental processes leading to the MFS aortic phenotype, we have recently shown a significant correlation between the expression levels of the extracellular matrix metalloproteinase (MMP) inducer (EMMPRIN) and MFS thoracic aortic disease severity [63,64].

## 2. Genetically Related Disorders in MFS Differential Diagnosis

Many phenotypes resembling MFS in terms of cardiovascular, ocular, or skeletal involvement, but not fulfilling the diagnostic criteria, have been described in the last few years. Interestingly, while some patients with these phenotypes were found to carry a mutation in the *FBN1* gene, other patients show causative mutations in other genes, probably belonging to the same pathway.

The genetic allelic disorders and common disorders in the differential diagnosis are listed below.

Neonatal Marfan syndrome (ORPHA:284979) represents the most severe form of MFS, manifesting during the neonatal period and generally associated with a poor prognosis. Symptoms include the classic Marfan syndrome characteristics, coupled with facial dysmorphism such as crumpled ears, loose redundant “senile” skin, flexed joint contractures, pulmonary emphysema, and progressive cardiovascular complications (i.e., dilatation of the ascending aorta and severe insufficiency of the mitral and/or tricuspid valve). This condition encompasses skeletal manifestations such as arachnodactyly, dolichostenomelia, and chest deformities. Currently, the prevalence of this autosomal dominant disease is still unknown [65]. Newborns are often preterm, SGA with median birthweight and lower birthweight centile when compared to controls [66]. The mutations that cause MFS affect the *FBN1* gene. In contrast to MFS, where mutations are generally inherited in an autosomal-dominant fashion, mutations causing neonatal MFS are frequently de novo and are usually found between exons 23 and 32 of the *FBN1* gene, known as the neonatal region [67].

Autosomal dominant Weill–Marchesani syndrome (OMIM # 608328) manifestations include microspherophakia, lens subluxation, short stature and brachydactyly, without any vascular involvement [68]. Interestingly, Weil–Marchesani syndrome’s phenotype can also encompass glaucoma [68]. The condition is due to a mutation in the *FBN1* gene; specifically, fibrillin-associated mutations causing the dominant form of Weill–Marchesani syndrome include specific substitutions and amino acid deletion affecting domain TB5, substitutions in the first hybrid domain, and a case of deletion of exons 9–11 resulting in the loss of domains TB1 to EGF4 [54,68]. All mutations lead to the dysregulation of TGFβ.

Autosomal recessive Weill–Marchesani (OMIM # 613195)—clinical features in this form can overlap autosomal-dominant Weill–Marchesani syndrome, but genes involved in the condition are: *ADAMTS10*, *ADAMTS17*, and *LTBP2* (described in one family, without Ectopia Lentis). In this regard, ADAMTS proteins play a role in the construction, durability, and fastening of microfibrils; they also contribute to the formation of specialized networks that consist of microfibrils and serve specific functions [51]. Furthermore, the Latent Transforming Growth Factor Beta-Binding Protein 2 (LTBP2) is a component of the extracellular matrix that interacts with microfibrils containing Fibrillin-1 [51].

Geleophysic dysplasia 2 (OMIM # 614185) is a rare skeletal dysplasia characterized by postnatal onset short stature (<3 standard deviation), delayed bone age, short limbs, and brachydactyly with markedly short tubular bones and relatively normal epiphyses, progressive joint contractures, progressive thickening of heart valves and facial anomalies (other than those commonly seen in MFS). In addition, hepatomegaly is also present. Tracheal stenosis and, in general, respiratory insufficiency are described in the literature and can become one of the most invalidating factors for the prognosis of these patients.

Acromicric Dysplasia (OMIM # 102370) is a condition overlapping Geleophysic Dysplasia 2 [69], as it shares skeletal and joint phenotypes. Mutations always affect the *FBN1* gene; they are all located in exons 41 and 42, and encode TGFβ-binding protein-like domain 5 (TB5) of the *FBN1*. Both syndromes are inherited in an autosomal dominant manner.

It is important to remember that Autosomal Recessive Geleophysic Dysplasia has also been described. Clinical features may overlap Geleophysic Dysplasia 2, but the causing gene is the ADAMTS-like protein 2 gene (*ADAMTSL2*) that is involved in TGFβ bioavailability.

Stiff skin syndrome (OMIM # 184900) is a rare, autosomal dominant cutaneous disorder with progressive, symmetric, sclerotic skin changes of the shoulders, hips, and thighs, joint contracture, and relatively short stature without the typical skeletal, ocular, and cardiovascular features of MFS [70]. All *FBN1* mutations known until now and related to the disease affect domain TB4 of Fibrillin-1, which contains the only integrin-binding RGD motif of the molecule [38]. The fibrotic phenotype is considered to be regulated by changes in the ability of *FBN1* to mediate integrin binding [71].

Marfanoid progeroid lipodystrophy syndrome (OMIM # 616914) is a rare condition with an estimated prevalence of <1/1,000,000 [72]. The main clinical features include poor weight gain since birth, postnatal lipodystrophy, muscle wasting, and generalized subcutaneous fat reduction leading to a progeroid appearance of the body in all subjects. Peculiar facial dysmorphisms, marfanoid habitus, hyper extensible joints, arachnodactyly, severe myopia (very common), ectopia lentis (less common), increased aortic root dilatation risk, and mitral valve prolapse can be present. Oligohydramnios, premature delivery, and craniosynostosis are described in some cases. In all affected subjects described until now, the syndrome arises due to a mutation in exon 64 in the C-terminus domain of the *FBN1* gene, leading to a premature stop-codon [73,74].

MASS phenotype (OMIM # 604308) is the acronym for Mitral valve–Aorta–Skeleton–Skin syndrome. The following are mandatory features: mitral valve prolapse, myopia, borderline and non-progressive aortic enlargement, and skin and skeletal systemic features with a systemic score ≥ 5 as defined in the revisited Ghent criteria. In several cases, the MASS phenotype occurs due to a mutation in the *FBN1* [75,76].

Bicuspid aortic valve (BAV): a common cardiac defect occurring in 0.5–2% of the general population. It can manifest alone or be associated with other congenital defects and syndromes, as in some cases of MFS. The *FBN1* pathogenetic variant was found in some affected individuals.

Mitral valve prolapse syndrome (OMIM # 157700). The prevalence is about 2 to 3% in the general population. It shares the characteristic features of MASS; some individuals carry the mutation in the *FBN1* gene. The systemic score is <5. In some cases, mild aortic root dilatation (Z-score < 2) is present [77].

Loeys–Dietz syndrome (LDS) (OMIM # 613795): this syndrome overlaps with the MSF in aortic root aneurisms and risk of dissection, skeletal features, and habitus. Nonetheless, some important differences in clinical features exist. Facial appearance is different: craniosynostosis, hypertelorism, bifid uvula, and cleft palate are present in many patients. Ectopia lentis is never present.

Arterial tortuosity is much more pronounced; aneurysms involve not only the aortic root, but can also spread to aortic branches and cerebral vessels. Additionally, cardiovascular manifestations are present at a younger age [78]. LDS patients often present cardiovascular features early in life, which is not the case for MFS [79].

Joint hyperlaxity can play a crucial role in determining prognosis. In severe cases, marked hyperlaxity leads to severe motor delay, severe kyphosis and scoliosis, recurrent joint dislocations, and cervical spine instability. Cervical spine malformation and/or instability can lead to severe complications; careful monitoring is required. On the contrary, in some patients, joint contractures are observed. Furthermore, hernias, thin translucent skin, poor wound healing, and atrophic scars are also frequently observed in LDS and not in MFS.

LDS can be caused by mutations in genes such as *TGFBR1*, *TGFBR2*, *SMAD2*, *SMAD3*, *TGFB2*, and *TGFB3* that play a part in the TGF-β signaling pathway.

The genes *TGFBR1* and *TGFBR2* are frequently involved in the mutation (respectively, 20–25% and 55–60% of cases). *TGFB2*, *TGFB3* and *SMAD2* and *SMAD3* are rarely identified (1–10%).

As yet, a strong genotype–phenotype correlation has not been established.

Nevertheless, mutations in *TGFBR1* and *TGFBR2* have been related to the previously described phenotype [80].

Patients with mutations in *SMAD3* have a strong predisposition to osteoarthritis; cardiovascular involvement is mild. Some authors defined the phenotype as “aneurysms-osteoarthritis syndrome” or LDS type III [81].

Beals syndrome (Congenital contractural arachnodactyly CCA) (OMIM # 121050) is a rare autosomal dominant connective tissue disorder (estimated prevalence < 1/10,000 worldwide) characterized by a Marfan-like appearance with crumpled ears, arachnodactyly, contractures, camptodactyly, muscular hypoplasia, and kyphosis/scoliosis (in approximately half of all affected individuals). Recent reports also mention aortic root dilatation, a finding previously thought to differentiate the condition from MFS. Likewise, ocular manifestations are unusual but possible [82]. Mutations in *FBN2* codifying for Fibrillin 2 protein were discovered to cause Beals syndrome. All mutations clustered between exons 24 and 35, which encode the calcium-binding epidermal growth factor-like (cbEGF) domains [42]. These pathogenic variants reduce the amount of Fibrillin-2 available to form microfibrils. Decreased microfibril formation reduces the elasticity of fibers, which leads to the symptoms of CCA [83].

Meester–Loyes syndrome (OMIM # 300989) is a very rare and new genetic condition characterized by facial features resembling MFS, joint hypermobility or contractures, and an elevated risk for early-onset thoracic aortic aneurysm and mitral and aortic valve insufficiency. The causative gene is *BGN*, mapped on the X-chromosome. *BGN* codifies for the biglycan protein of the ECM. The syndrome recognizes an X-linked mode of inheritance, but also carrier females can manifest symptoms, and their phenotype ranges from unaffected to fatal aortic dissection [78].

Ehlers–Danlos syndrome (EDS). The Ehlers–Danlos syndromes represent a heterogeneous group of inherited connective tissue disorders characterized by joint hypermobility, increased skin elasticity, and tissue fragility. Recognizing the genetic and clinical diversity of these conditions has prompted the development of various classifications over time. The “Villefranche Nosology” was established in 1998 [84]. In the following years, with the aid of new biochemical and genetics technologies, a whole spectrum of novel EDS subtypes was described, leading to overcoming the Villanche Nosology. Recently, a new classification scheme acknowledging 13 distinct subtypes of EDS has been proposed, reflecting an evolution in our understanding of the syndrome. This approach encompasses all phenotypes exhibiting the core clinical features of EDS: joint hypermobility, skin hyperextensibility, and tissue fragility [85]. Depending on which gene is involved, genetic transmission is autosomal dominant (the most frequent) or autosomal recessive.

EDS syndrome subtypes can resemble MFS when it comes to aneurism risk, joint hypermobility, and hyperextension skin.

In the following paragraphs, the EDS subtypes mainly cited in differential diagnosis with MFS will be described.

EDS, classic type (OMIM # 130000): an autosomal dominant condition characterized by skin hyperextensibility, delayed wound healing, velvety smooth skin, and joint hypermobility. More than 90% of patients harbor a heterozygous mutation in one of the genes encoding type V collagen (*COL5A1* and *COL5A2*) [86]. A genotype–phenotype correlation has been tested. When specific mutations are present (heterozygous arginine-to-cysteine substitution mutations c.934C>T, p.Arg312Cys; c.1720C>T, p.Arg574Cys and c.3277C>T, p.Arg1093Cys), vascular fragility and risk for vascular rupture is high, mimicking the vascular type EDS (see below) [87]. In addition, a classical-like EDS has been listed in the 2017 classification: the transmission manner is autosomal recessive, and the involved gene is *TNXB*.

EDS cardiac-valvular type (OMIM # 225320) is an autosomal recessive disorder and the involved gene is *COL1A2*. The main features are severe and progressive cardiac-valvular problems (aortic valve, mitral valve) and general EDS-like skin involvement (thin skin hyperextensibility, atrophic scars, and easy bruising). Some patients have chest deformity (especially pectus excavatum) and joint hypermobility (generalized or restricted to small joints).

EDS, vascular type (OMIM # 130050) is autosomal dominant disorder, caused by mutations in the *COL3A1* gene. Diagnostic criteria include a family history of the disorder, arterial rupture or dissection in individuals under 40 years of age, abnormal rupture of the sigmoid colon, or spontaneous pneumothorax accompanied by other EDS features. Additional characteristics include translucent skin through which veins are easily seen, a propensity for bruising, wide and abnormal scarring, and distinct facial features such as protruding eyes and a tight or “pinched” appearance. The tendency for aneurysm and/or dissection involves any medium to large muscular artery throughout the body. Tissues can be extremely friable, often contributing to surgical catastrophe; endovascular procedures have an increased risk profile due to the delicate vasculature [88]. The disease can also lead to major neurological complications, including carotid-cavernous fistulae aneurysms of the Circle of Willis [85].

Hypermobile EDS (OMIM % 130020) is typically inherited in an autosomal dominant manner, though the specific gene involved remains unknown. Diagnosis is largely based on clinical presentations. Symptoms can range from asymptomatic joint hypermobility through “non-syndromic” hypermobility with secondary manifestations to severe generalized hypermobility. These presentations can vary by age and gender.

EDS, kyphoscoliotic form (OMIM # 225400), is an autosomal recessive disorder caused by a biallelic mutation in *PLOD1* gene or biallelic mutations in *FKBP14* in very few cases, which has been verified before now [89]. It is characterized by fragile, hyperextensible skin, thin scars, easy bruising, generalized joint laxity, and severe muscle hypotonia at birth. Other features include progressive kyphoscoliosis, present at birth or developing within the first year of life, and increased risk of globe rupture due to scleral fragility. Severe kyphoscoliosis may lead to respiratory complications, and patients are also at risk for the rupture of medium-sized arteries [85].

Brittle cornea syndrome (OMIM # 614170, # 229200) is, to date, a very rare autosomal recessive condition. The disorder is due to a mutation in *PRDM5* or *ZNF469* genes. The main features are ocular alterations such as having a thin cornea with a high risk of corneal rupture, progressive keratoconus, high myopia, and retinal detachment. Other common clinical features are marfanoid habitus with scoliosis, arachnodactyly, hypermobility of distal joints, pes planus, hallux valgus and mild contractures of fingers (especially the fifth), as well as soft, velvety, and translucent skin. The phenotypic spectrum of brittle cornea syndrome appears in an extremely similar, if not identical manner, in patients with mutations in either *ZNF469* or *PRDM5*, suggesting that the two genes act within the same developmental pathway. The authors found a carrier phenotype in the majority of heterozygous individuals examined [90,91]. Blue sclerae, mildly reduced central corneal thickness, and small joint hypermobility were present in the large majority of those identified with heterozygous *PRDM5* mutations. It is important to consider that heterozygote status might confer a higher risk for developmental dysplasia of the hip, as well as visual impairment or deafness. On the contrary, other clinical features such as hearing loss, easy bruising, and laxity of the large joints were detected only in affected individuals (individuals with biallelic mutations) [91].

Arterial tortuosity syndrome (OMIM # 208050) is a rare autosomal recessive connective tissue disorder characterized by the twisting and elongation of large and medium-sized arteries. Patients with ATS face cardiovascular issues including stenosis of the arterial and pulmonary valves, increased risk for aortic aneurysms and vascular dissection, abnormal origin of aortic branches, and vasomotor instability. Arterial tortuosity syndrome also shows additional signs shared with other connectivopathies, including soft/velvety/hyper extensible skin, mild dysmorphic facial features (hypertelorism, high palate, bifid uvula, micrognathia), hernias and joint hypermobility and instability [92]. The gene involved is *SLC2A10* [93].

Classical Homocystinuria (OMIM # 236200) is a rare autosomal recessive disorder (incidence between 1/335,000 and 1/200,000) of the methionine metabolism, manifesting with an abnormal accumulation of total homocysteine in the blood, increased excretion of homocysteine in urine, an elevated methionine blood concentration along with a decrease in plasma cystathionine. The genetic defect is in the cystathionine beta-synthase gene (*CBS*), resulting in a deficiency of cystathionine synthetase enzyme activity [94]. The clinical features of untreated homocystinuria usually manifest in the first or second decade of life and include myopia, ectopia lentis, mitral valve prolapse, accelerated skeletal growth, osteoporosis, skeletal anomalies resembling MFS (body habitus, pectus deformity, kyphoscoliosis, hernias), thromboembolic events and variable intellectual disability. The classic disorder can have two distinct phenotypes: a milder responsive form (vitamin B6) and a more severe and form nonresponsive to pyridoxine [95]. Pyridoxine is a cofactor for the CBS enzyme, and can aid in converting homocysteine to cysteine. The milder form of homocystinuria, which has been identified in few individuals, consists of increased plasma homocysteine and the risk for thrombotic events in young adulthood, as well as the absence of any other systemic manifestations observed in classic homocystinuria [40].

Stickler syndrome (OMIM # 108300) is a clinically and genetically heterogeneous disease that was first described by Stickler et al. [96]. The incidence among newborns is approximately 1:7500–1:9000. Stickler syndrome is characterized by ocular, skeletal, auditory, and orofacial abnormalities [97]. The ocular finding includes myopia (90% of cases) and congenital vitreous abnormalities leading to an increased risk of retinal detachment and hemorrhage (69% of cases). Most patients also have joint pain, usually secondary to degenerative disease (osteoarthritis). In 84% of patients, orofacial anomalies such as flat midface, depressed nasal bridge, micrognathia, cleft palate, and Pierre–Robin sequence have been described. Some degree of hearing loss is present in 70% of patients [96]. At present, six subtypes of Stickler syndrome have been discerned, and both ocular and genetic findings subclassify them. The involved genes are *COL2A1*, *COL11A1*, and *COL11A2* for the autosomal form and *COL9A1*, *COL9A2*, or *COL9A3* for the autosomal recessive form of the disease. Mutations in the *COL2A1* gene cause type 1 Stickler disease, the most common form, accounting for 80–90% of the total cases 74. In types I and II, the ocular finding is frequent; in type III, the systemic features are present without ocular features.

Heritable Thoracic Aortic Disease (HTAD, OMIM # 607086) is a condition involving a genetic variant that significantly increases the risk of thoracic aortic diseases. The incidence is about 10.4 per 100,000 people per year, with a strong genetic component, with up to 20% of affected individuals having a positive family history [98]. The implicated genes and their associated proteins have been found to act on a diverse variety of functions linked to transforming growth factor beta (TGF-β), signaling pathways, disruption of the vascular smooth muscle cells, and the disruption of extracellular matrix homeostasis [99]. Mutated genes confer a high risk for thoracic aortic aneurysm and dissection; the penetrance is incomplete and age-related, and the expression is instead variable [100]. According to the limited data available thus far, children usually do not manifest aortic dilatation/aneurysm. In 2018, a panel of 53 genes was proposed to test syndromic and non-syndromic patients with thoracic aortic aneurysms [101]. Most of the genes are inherited in an autosomal dominant manner (*n* = 37); 11 genes showed autosomal recessive inheritance (*ADAMTS10*, *B3GAT3*, *CBS*, *COL9A1*, *COL9A2*, *COL18A1*, *EFEMP2*, *GJA1*, *PLOD1*, *PLOD3*, and *SLC2A10*); 4 genes yielded X-linked recessive inheritance (*COL4A5*, *FLNA*, *MED12*, and *UPF3B*) and 1 gene showed X-linked dominant inheritance (*BGN*).

Autosomal dominant polycystic kidney disease (ADPKD, OMIM # 173900, # 173910) shares an increased risk of aortic root aneurysms with MFS. The genes involved are *PKD1* and *PKD2*. The process of aortic aneurysm formation in ADPKD has not yet been completely understood; it is speculated that PKD1 haploinsufficiency leads to the upregulation of TGF-ß signaling [99,102].

Chromosome 16p13.3 duplication including *MYH11* gene—chromosomal deletions or reciprocal duplications of the 16p13.1 region have been linked to several neuropsychiatric conditions such as autism, schizophrenia, epilepsy, and attention-deficit/hyperactivity disorder (ADHD). Kuang et al. [100] screened 765 patients with adult-onset TAAD for CNVs and identified a recurrent 16p13.1 duplication in 1% of TAAD cases compared with 0.09% of controls. They concluded that (A) 16p13.1 duplications are associated with an adult-onset cardiovascular disorder and also in the absence of significant neuropsychiatric abnormalities, and (B) the presence of the 16p13.1 duplication confers a risk for thoracic aortic disease even if the penetrance is not complete. The decreased penetrance of TAAD associated with the duplication suggests that other risk factors are required for the expression of the clinical phenotype (other genetic variants, such as another CNV, a single gene mutation, or any other known risk factors for TAAD such as uncontrolled hypertension) [100].

Klinefelter syndrome (karyotype 47,XXY) is a well-known chromosomopathy with a prevalence of 1/600–1/800 male births, affecting male sexual development and fertility. It can share with MFS skeletal habitus, low muscle mass, and the presence of mitral valve prolapse (until 55% of cases) [103].

Turner syndrome (Karyotype 45,X) is a well-known chromosomopathy with a prevalence of 1–5/10,000 female births, affecting female sexual development, fertility, and stature (short proportionate stature). Turner syndrome patients are prone to various heart conditions such as bicuspid aortic valve, aortic coarctation, and aortic dilation or dissection. Reports suggest that aortic dilation can occur in up to 40% of Turner syndrome cases [104].

Fragile X-linked syndrome (FXS) is a well-known X-linked recessive disorder with a prevalence of 1/2000 males and 1/4000 females. In affected males, intellectual disability, behavioral abnormalities, attention-deficit/hyperactivity disorder (ADHD), autistic-like behavior, and speech delay are very common. Seizures have been described in 20% of cases.

The physical characteristics of FXS include an elongated face, broad forehead, high palate, prominent ears, hyper extensible finger joints, flat feet and macro-orchidism that becomes more prevalent with age [105]. In addition to commonly recognized characteristics, patients may have a variable presentation of connective tissue alterations resembling MFS. Mitral valve prolapse and aortic root dilatation can affect adult life. The disorder is more likely to severely affect the male population than females. The genetic abnormality behind FXS is a “full mutation” in the *FMR1* gene, which is marked by an expansion of CGG trinucleotide repeats (over 200) and unusual methylation of the gene [105].

Lujan–Fryns syndrome is another X-linked recessive intellectual disorder, probably rarer than Fragile-X syndrome (the prevalence is unknown). Common clinical features include macrocephaly, tall stature, long face with maxillary hypoplasia, high narrow palate, hyper nasal speech, long hands and digits, joint laxity or contracture, muscle wasting and hypotonia, mild-to-moderate intellectual disability, behavioral abnormalities, autistic-like behavior, dysgenesis of the corpus callosum and seizures [106]. The cardiovascular system can be involved, with the development of the following defects: atrial septal defect, ventricular septal defect, and only rarely, ascending aortic aneurysm.

A mutation in the *MED12* gene, which plays a critical and central role in the transcription of RNA polymerase II, has been identified in some patients. As a multiprotein complex, this mediator regulates cell growth, development, and differentiation signals. It is implicated in a protein network involved in extraneuronal gene silencing and other functions, such as the direct suppression of Gli3-dependent Sonic hedgehog signaling [106,107]. Until now, no interaction between the *MED12* gene and TGFB pathway genes has been described.

Marfanoid habitus with intellectual developmental disorders—rare syndromes with an intellectual developmental disorder and marfanoid habitus are described in the literature, and are often so rare that they have not been named yet and are only recognized by these two clinical features, along with the gene mutated in the affected individuals [108,109,110].

All pertinent information discussed above regarding differential diagnoses for Marfan syndrome has been comprehensively synthesized in Supplementary Tables S1 and S2. Supplementary Table S1 contains detailed information on disorders associated with FBN1 gene mutations other than Marfan syndrome, while Supplementary Table S2 presents a comparative analysis of genetically related disorders, included in the differential diagnosis of Marfan syndrome.

## 3. Management and Treatment of MFS Patients

The optimal management approach involves a collaborative effort amongst a diverse team of specialists. This includes a clinical geneticist, a cardiologist, an ophthalmologist, an orthopedist, and a cardiothoracic surgeon, among other specialists as needed.

Based on the recent data in the literature and our personal experience at the Cardiovascular Genetic Centre, IRCCS Policlinico San Donato in Milan, we propose the following evaluation.

### 3.1. Cardiovascular System

During the initial diagnosis, we recommend 2D-transthoracic echocardiography (2D-TTE) in the assessment and follow-up of the aortic root and the proximal ascending aorta, the current gold standard for clinical monitoring and evaluation [104]. According to the current convention, measurements are obtained from the parasternal long-axis view at the end-diastole.

The Z score is calculated with the Roman formula [79], according to recommendations of the Marfan Foundation. The aortic Z score, corrected for body height, is validated and widely used in clinical practice for regression analysis [111]. Aortic dilatation is defined according to Ghent 2010 criteria [4].

Imaging must be performed in accordance with the established guidelines; obtained values must be corrected for age, body surface area (BSA), and gender. Follow-up should be arranged based on the patient’s peculiar clinical features and history (known aneurysmal lesion or previous arterial dissections). A complete vascular imaging of the thorax and abdomen (neck to pelvis) is also suggested from the age of 18 years by CT scan or MRI once every 2 to 5 years [104,112].

The CT and MRI scan results allow clinicians to understand the morphology of the entire aorta and peripheral vessels. The CT has the best special resolution, but unlike the MRI, it has the burden of ionizing radiation. Hence, the choice between a CT and an MRI largely depends on local expertise and availability, with the overarching aim being to minimize radiation exposure whenever possible.

There is a general consensus that the aortic diameter should be measured, in both imaging techniques, with the inner-to-inner convention during the end-diastolic phase [104].

The normal aortic diameter is influenced by a number of factors, including patient age, sex, and body size; location of aortic measurement; method of measurement and the robustness and type of imaging methods used. Furthermore, studies have highlighted that the diameter increases by 0.12 to 0.29 mm/y at each level measured by CT in both men and women aged 18 years of age and over [113]. MFS is influenced by the over-regulation of several aortic growth factors, and therefore, we recommend 2D-TTE every 6 months, in the absence of rapid increased aortic root diameter in child or adult patients (in these cases the control rate could be shortened, depending on clinical judgment) [114].

Annual imaging is recommended for patients with MFS if the stability of the aortic diameter is documented. Further imaging is suggested in the presence of a maximal aortic diameter of 4.5 cm or greater, or in the case of significant aortic diameter growth from baseline. An abdominal CT scan or MRI is recommended every 2–3 years, whereas complete imaging of the vascular neck tree and CNS is recommended once every 3–5 years.

According to von Kodolitsch et al. [111], we must not forget that MFS also affects the heart valves, especially the left-sided valves. Their involvement accounts for the majority of clinical symptoms in an age-dependent manner. Therefore, valve evaluation by 2D-transthoracic echocardiography should be similarly included in the clinical surveillance and decision-making process [111].

Recent studies raise the suspicion that the incidence of cardiomyopathy and arrhythmia in MFS patients is not so rare. For this reason, we recommend paying attention to these factors when planning the follow-up [111].

### 3.2. Assessment of Aortic Distensibility

Numerous studies have proven that aortic stiffness is also able to predict, throughout the whole aorta, the development of aortic luminal growth and dilatation [115,116,117]. The carotid–femoral pulse wave velocity (PWV) is currently the gold standard for the non-invasive assessment of arterial stiffness through arterial tonometry. PWV evaluation has a proven predictive value for cardiovascular morbidity and mortality [115].

Moreover, the ascending aorta, along with other large elastic arteries, serves a critical function in regulating blood pressure and peripheral blood flow. They mitigate the pulsating output from the left ventricle, ensuring the heart’s cyclical, intermittent, and discontinuous pumping activity leads to a steady flow of blood [118]. As shown in the literature, accelerated arterial stiffening is associated with aneurysmal dilatation of the ascending aorta, especially when this is associated with altered Fibrillin-1 synthesis, therefore suggesting that the MFS phenotype is associated with increased arterial aging [116]. So far, the assessment of central pulse wave pressure carried out in our studies has highlighted impairment in the pediatric MFS population, with exacerbated PWV in patients with increased aortic dilation within a year of follow-up [119].

Furthermore, although the ascending thoracic aortic aneurysm is associated with a pulsus tardus, this characteristic does not influence the viscoelastic properties of the total aorta after the insertion of a rigid prosthesis in the ascending section [118].

### 3.3. Aortic Surgical Treatment

The definition of a dilated ascendending aorta is related to a dimension > 4.0 cm, but elective aortic root replacement should be considered in asymptomatic patients with a maximal aortic diameter between 45 and 50 mm, following the American Heart Association (AHA) recommendations. Surgical repair of the dilated aortic root/ascending aorta for patients with MFS is usually performed at a threshold of an external diameter of 5.0 cm; however, in the presence of factors such as the rapid growth of the aortic diameter (greater than 0.5 cm/year), a family history of and the presence of significant aortic regurgitation, surgical correction is recommended in patients with aortic diameter below than 5.0 cm [120]. In the present era, valve-sparing aortic root replacement, particularly following David’s re-implantation surgery technique, is the preferred solution, especially for young patients (avoidance of lifelong anticoagulation during an active lifestyle and with the perspective of pregnancy for female patients). The timing of the surgery depends primarily on the size of the aorta, its growth rate and the presence of valve regurgitation. However, the decision about timing and the surgical approach must be carefully shared with the patient. Mechanical valve replacement is a more durable option, but exposes the patient to the risk of iatrogenous bleeding and thromboembolic hazard. Conversely, valve-sparing surgery carries a potential lifetime risk of valve dysfunction and subsequently the possibility of further surgery [121,122]. The concept of “personalized surgery” introduces another option in aortic root surgery for Marfan patients, referred to as the PEARS (Personalized External Aortic Root Support) technique.

First introduced in 2013, PEARS is an external device that supports the ascending aorta, avoiding its replacement. The term “personalized” refers to the fact that the device is manufactured as a 3D reconstruction of an individual patient’s aorta. The device, made of medical grade polymer fabric, wraps around the patient’s aorta, and its placement does not usually require cardiopulmonary bypass and preserves the blood–endothelial interface. PEARS is not indicated in the presence of more than mild aortic regurgitation, and is usually performed in patients with a smaller aortic diameter than those who undergo valve-sparing surgery [123].

### 3.4. Ocular System

A primary and detailed ophthalmologic diagnosis is needed to evaluate the refraction, intraocular pressure, lens status, peripheral retina status, and changes in the optic nerve.

Afterward, an ophthalmologist with expertise in MFS should manage ocular manifestations annually.

In the case of lens subluxation, eyeglasses represent the primary management approach to correct the refractive error. In the case of anterior lens dislocation, the primary treatment consists of an extraction of the lens [124]. Surgery is recommended in the following cases: inability to gain a proper visual acuity, high risk of amblyopia in the younger population, posterior dislocation of the lens towards the vitreous cavity, anterior dislocation of the lens with or without secondary glaucoma, imminent dislocation of the lens, lens-induced glaucoma or uveitis and cataract. It is important to consider that in these patients, the extraction of sub-luxated is challenging; moreover, complications such as the loss of the capsular bag, vitreous disturbance, and endothelial cell damage are frequent in the presence of zonular weakness and lens instability [125]. The intraocular lens can be implanted after puberty [124].

Children at high risk for amblyopia need timely correction assessment. Corneal refractive surgery (laser keratotomy) is not recommended for most patients with MFS, as the cornea is markedly flat in these cases. MFS individuals with myopia can undergo laser surgery only in the absence of lens dislocation; on the contrary laser ablation is not recommended because it may worsen the dislocation.

The management of glaucoma starts with antiglaucoma medication. Systemic beta-blockers for cardiovascular diseases have a minimal effect on lowering intraocular pressure when a topical beta-blocker is administered. Consideration of the lens position is needed prior to glaucoma surgery in patients with MFS. If the lens is normal, minimally invasive glaucoma surgeries or non-penetrating deep sclerotomy are preferred as first-line interventions because of the higher exposure to hypotony-related complications in incisional surgeries, and risk of further complications related to lens subluxation in the postoperative period [125].

### 3.5. Skeletal System

At the initial diagnosis, an orthopedic evaluation is required to identify all the skeletal markers expected in MFS.

An X-ray of the pelvis is done to detect the medial protrusion of the femoral head (protrusio acetabuli); being a frequent skeletal feature in MFS, a prolonged acetabular protrusion may result in secondary osteoarthritic changes in the hip joint [126].

In adult patients, a lumbar MRI is done to detect dura mater ectasia.

After a preliminary assessment, we suggest an orthopedic evaluation once a year or more frequently in childhood. Skeletal complications requiring close monitoring and orthosis devices or surgical intervention are scoliosis, kyphosis, pes planus, and pectus excavatum.

The adult-linked complications worth pharmacological, FKT, or surgical treatment are joint degeneration and pain, joint instability, spine deformity, and hallux valgus.

Pectus deformity may further compromise respiratory function as well [127].

Since many MFS patients have a high palate with dental crowing, dentistry follow-up with orthodontia is suggested.

Moreover, dental care is strongly recommended for all ages due to the high risk of periodontal disease in connective tissue disorders and cardiac valve infections.

Because of the increased risk of osteoporosis since childhood, in the MFS population, we recommend L1–L4 vertebrae level, femoral neck, and whole-femur dual-energy X-ray absorptiometry (DXA) every 2 years.

The bone mineral measurement must be expressed as real bone mineral density (BMD in g/cm^2^ and Z-score), according to the International Society for Clinical Densitometry (ISCD) recommendations, because patients with MSF are taller than the general population, and so bone mass measurement should be adjusted for height [128].

### 3.6. Respiratory System

Because of the high risk of pulmonary function alterations due to chest and spine deformity, restriction lung disease, and upper lobe blebs, we recommend spirometry plus DLCO every year. The detection of sleep apnea needs investigation through a questionnaire or through polysomnography. According to studies, the treatment of sleep apnea in patients with Marfan syndrome is the same as in the general population (weight reduction in those patients with overweight and nasal continuous positive airway pressure—CPAP), and is successful in improving fatigue and quality of life. The effects sleep apnea has on other organs and systems as well as on the quality of life have not been studied in Marfan syndrome, but could be very relevant [129]. It is important to note that individuals with sleep apnea that is unrelated to Marfan syndrome appear to have a higher cardiovascular risk, showing a higher prevalence of hypertension, stroke, and arrhythmia. The relationship between sleep apnea and aortic root growth in the MSF population is still under debate [130].

### 3.7. Psychological Support Activities

Psychological support is provided to patients (young and adults) belonging to the center, coming from all over the national territory, for preliminary evaluation or regular half-yearly and/or annual follow-up. Considering pediatric patients, psychological counseling could be carried out indirectly thorough parental interviews. Consultants aim to improve patients’ treatment compliance and identify the presence of MFS-related psychological distress. In these cases, psychologists provide individual counseling and/or psychotherapy sessions, as well as adequate psychological or psychiatric referrals outside the center for patients who reside far from it. The psychologist also organizes monthly self-help groups for patients and their families to help them feel less isolated and lonely (which is frequently common in patients with Marfan syndrome) and to create a space for sharing their life experiences that enhances the elaboration of their personal history related to the disease. The service we recommend should provide: (a) psychological counseling; (b) psychological counseling to patients and couples that are planning a pregnancy; (c) psycho-educational activities of cognitive behavioral imprint; (d) the presence of medical professionals when communicating the diagnosis to the patients; (e) parental counseling interviews; (f) self-help groups.

In conclusion, the management of MFS is summarized in Table 1.

### 3.8. Pharmacological Treatment

Comparing the various groups of anti-hypertensive medications that have been examined for their prophylactic effectiveness on aortic dilatation (beta-blockers (BB), Angiotensin receptor blockers (ARBs) with and without baseline BB therapy, and ACE inhibitors), medical therapy in MFS should be established according to patient tolerance and various risk factors, including age and family history of aortic dissection. According to Singh and Lacro [131], it is recommended that those patients diagnosed with aortic root dilatation should receive therapy with adequate doses of either a beta-blocker or ARB, and if severe, a combination of these therapies should be considered [131].

For Angiotensin receptor blockers (ARB), favorable effects leading to a reduction in the rate of progressive aortic root dilation in patients with MFS have been reported. Among ARBs, losartan is the most used, as mentioned below.

As regards the role of losartan, studies show that in the mouse model of MFS, increased TGF-β signaling appeared to play a pivotal role in different phenotypic features of MFS (i.e., progressive aortic root dilatation, lung defects resulting in bullae formation and failed muscle regeneration) [132]. In vivo studies have emphasized how abnormal changes in the aortic wall and the gradual enlargement of the aortic root can be mitigated or even averted by systemically administering a TGF-β-neutralizing antibody or an angiotensin II-receptor blocker such as losartan. This latter drug is an antihypertensive medication recognized for its ability to inhibit TGF-β signaling [133]. The therapeutic benefits relevant to MFS involve a decrease in the rate of expansion of the aortic root’s diameter. While losartan does not halt aortic growth completely, it specifically curtails the abnormal rate of enlargement in aortic segments that have already reached the necessary size to meet the physiological demand for blood flow to the tissues [133]. In the last decade, losartan has emerged as a potentially effective novel treatment strategy, as it is able to inhibit TGF-β signaling and consequently can prevent aortic root dilatation in MFS mouse models [134]. Furthermore, losartan treatment could be attributed to the selective blocking of angiotensin-II-type 1 receptor within the renin–angiotensin–aldosterone system, and reduces principal TGF-β signaling in the aorta [135]. Scientific evidence reports that losartan slows down the aortic root dilatation rate [133].

Clinical randomized trials show the beneficial effects of losartan in adult MFS patients [136], highlighting the decreased average rate of aortic root dilation in the losartan group, independent of factors such as age, sex, blood pressure, aortic root size, the presence of an FBN1 mutation, and concurrent use of β-blockers. Also, losartan is significantly associated with a reduced aortic arch dilatation rate in patients who underwent aortic root surgery [136].

Beta-adrenergic-receptor antagonists (beta-blockers (BB)) have been the first treatment for preventing aortic complications since the medical intervention was introduced in the 1970s. In the mid-1990s, a randomized trial demonstrated that prophylactic beta-adrenergic blocking with propranolol slowed the rate of aortic dilatation and the onset of aortic complications in the MFS population. As a result, beta-blockers became the standard preventive treatment [137].

Current BB in use include propranolol, atenolol, and metoprolol [138], of which atenolol is the drug of choice, being more cardioselective and having a longer half-life than propranolol, with fewer side effects. Dosage is adjusted according to heart rate: the optimal target is keeping a 60 to 70 bpm rate at rest and lower than 100 bpm after submaximal exercise [138].

The BB side effects include bronchospasm, exercise intolerance, fatigue, depression, and first- and third-degree heart block [139]. Moreover, in a recent meta-analysis, it was reported that there was no evidence of clinical benefits derived from long-term beta-blockade in people with MFS [138].

Since 2007, treatment with inhibitors of Angiotensin converting enzyme (ACEI) have been demonstrated to reduce aortic root dilatation in patients with Marfan syndrome [62,79,133]. However, when compared to beta-blockers, they do not significantly attenuate aortic growth velocity (AGV) [140], though their superiority in improving aortic distensibility and stiffness with an associated slower rate of aortic growth has been demonstrated [141]. Similarly to ARBs, this class of drugs may prevent or delay the phenotypic expression of MFS antagonizing TGF-β and slowing or even decreasing defragmentation of the elastic fibers of the aorta [142]. They also reduce angiotensin II levels that are associated with cystic medial degeneration contributing to aortic rupture in MFS [143].

To date, the ACEs in use are perindopril and verapamil. As regards ACEs’ side effects, their use is contraindicated during pregnancy because of potential toxicity to the fetus.

## 4. Specific Precautions for MFS Patients: Physical Activities and Pregnancy

### 4.1. Physical Activity

Endurance sports can be of great value in controlling body weight, blood pressure, and fitness. The cardiologist should adjust the physical activity level, both in children and adults, considering the evaluation of aortic dimensions and valve function. In patients with aortic dilatation/aneurysm, we recommend low static dynamic sports such as swimming, walking, running and cycling, without participating in competitive activities. However, any sports or activities involving sudden isometric or static maneuvers, or those causing a substantial rise in arterial blood pressure, such as weightlifting, competitive football, basketball, handball, and tennis should be avoided [112]. As a general health guideline, all patients should be encouraged to abstain from smoking and substance abuse.

### 4.2. Pregnancy

Being the most common aortic diseases with a genetic basis similar to LDS, EDS, and bicuspid aortic valve, pregnancy in MFS is considered a risk factor for developing aortic dissections and arrhythmias, cardiac arrests, cardiomyopathy, and heart failure [144,145]. Studies have proven that the risk for aortic dissection (type A or B) is eight times increased in pregnant MFS women when compared to never-pregnant MFS ones [145]. The risk is especially high in the immediate post-partum period; this is probably due to the increased blood volume and cardiac output, and due to the hormonally mediated loss of elastic fibers in the aortic wall [146].

Specifically, the decreased tissue elasticity in MFS may compromise the vascular system’s ability to sustain the key physiological changes of pregnancy [147].

This is particularly apparent in the third trimester, a period when the majority of cardiac events are reported. This coincides with the peak of physiological stress in pregnancy, where there are substantial increases in various cardiac parameters. Specifically, cardiac output can increase by 40%, stroke volume escalates, and blood volume can increase by 30–40%. The heart rate also surges by 10–20 beats/minute in the first two trimesters, peaking at around the 32nd week of gestation with an intravascular volume spike of up to 50% [148].

Studies also show that, compared to controls, MFS patients have a high risk of preterm delivery, due to cervical incompetence related to the abnormal connective tissue, which is very frequent during pregnancy [144]. It is also likely that MFS women experience Intra-Uterine Growth Restriction (IUGR) and Small for Gestational Age (SGA) newborns [144].

As a matter of fact, pregnancy can be dangerous for women with MFS, resulting in increased maternal and fetal mortality and morbidity [148]. Preconception care is recommended, according to the literature [149]. Counseling with a pregnancy heart team with specialized cardiologists, clinical geneticists and obstetricians is useful in order to evaluate the aortic risk, define the reproductive genetic risk, and properly monitor the pregnancy, including the discussion of invasive prenatal diagnostic testing and pre-implantation genetic diagnostics.

All women who intend to proceed with a pregnancy should undergo imaging of the entire aorta before it [112].

Patients with an aortic diameter of ≥40 mm or those exhibiting progressive dilation should undergo transthoracic echocardiography (TTE) examinations every 4–6 weeks. For those with a normal-sized aorta, these assessments should be performed in each trimester [150]. According to the American guidelines (ACCF/AHA), pregnant women with aortic dilation are recommended to have monthly or bi-monthly echocardiographic measurements of their ascending aortic dimensions during pregnancy and for the initial weeks following delivery [151].

Women who have undergone aortic dissection should be advised not to attempt pregnancy, whereas ones with aortic root ≥ 45 mm are recommended for prophylactic aortic root replacement prior to pregnancy [112].

In women who decide to proceed with the pregnancy regardless, a planned cesarean section is suggested to avoid labor.

Regarding modes of delivery, cesarean section rates are considerably higher in this population. The 2010 American Guidelines suggest it as a preferred mode of delivery because MFS is a severe illness. However, patients may develop several complications related to the surgery: they have a fivefold increased risk of venous thromboembolic disease (VTE) and an increased risk of DIC when compared to controls [144].

Additionally, it is worth mentioning that in women with a prior diagnosis of MFS, a low incidence of aortic complications during pregnancy is reported in the presence of an initial diameter root ≤ 45 mm of the aortic, and with appropriate follow-up during pregnancy [145].

#### 4.2.1. Medical Therapy during Pregnancy

Research has indicated that the use of ß-blockers can slow aortic root expansion and considerably decrease the occurrence of aortic dissection and mortality [150]. While these results need further exploration in the context of pregnancy, current data suggest that the preventive use of ß-blockers during pregnancy is clinically sound. During pregnancy, it is recommended to use selective ß receptor blockers, with dosage adjusted to reduce heart rate by a minimum of 20 bpm, while closely monitoring for intrauterine growth restriction [152,153,154]. ARBs should be avoided during pregnancy due to potential harm to the fetus [155]; they should be replaced with ß-blockers once contraception is stopped and pregnancy is being planned. All pregnant women with MFS are advised to maintain strict blood pressure control. For acute aortic dissection during pregnancy, nitroprusside usage may lead to fetal thiocyanate toxicity; hence, using nitroglycerine or hydralazine in conjunction with ß-blockers to manage blood pressure is preferred during gestation [150].

#### 4.2.2. Prenatal Testing

Prenatal and/or preimplantation diagnoses become available once a pathogenic or likely pathogenic variant in FBN1 has been identified in the proband. The choice to pursue these methods is always a shared decision made by the couple. Currently, the routinely used procedures for prenatal diagnosis include chorionic villus sampling (CVS) and amniocentesis, both of which are performed under ultrasound guidance. CVS is carried out between 10 and 12 weeks of gestation, while amniocentesis is performed between 14 and 20 weeks of gestation. Both techniques carry a 1% risk of miscarriage.

An alternative to prenatal diagnosis can be preimplantation genetic diagnosis, a method that combines in-vitro fertilization treatment with the genetic analysis of single cells removed from embryos. This technique allows for the testing of the specific genetic alteration before embryo transfer and implantation. The oocytes are retrieved following ovarian stimulation and fertilized in the laboratory using either in vitro fertilization or intracytoplasmic sperm injection. Single cells are then retrieved from each developing embryo for DNA analysis. Unaffected embryos will then be transferred into the uterus, while affected embryos are, with the couple’s consent, humanely discarded [156].

Prenatal testing often comes with significant psychological implications for the couples. The decision to test and the potential subsequent diagnosis can be emotionally challenging, leading to heightened anxiety and stress. Psychological counseling can help in offering comprehensive information about testing options and aids in managing these emotional responses, thereby facilitating the decision-making process. In the event of a positive diagnosis, the counseling should extend to include guidance on understanding and preparing for life with a child affected by MFS, with an emphasis on developing resilience and effective coping strategies. Emotional support should also be readily available for siblings, extended family members, and close friends who are implicated in the care of the patient.

## 5. Conclusions

All the aspects explored in our review indicate that we are probably still far from having complete knowledge and understanding of the pathology of MFS and its mechanisms.

For this reason, it is essential to adopt a multidisciplinary approach to accurately screen the MFS population at diagnosis and to better plan health care management. The wide-ranging approach we use at the Cardiovascular Genetic Centre, IRCCS Policlinico San Donato, may represent an initial step towards a more comprehensive involvement of the patients and their families throughout the management of this life-long disease, considering that little evidence has been produced in terms of patient advantages related to a multidisciplinary evaluation and follow-up, meaning we are thus lacking in personalized and tailored treatments.

Moreover, the challenge for the future is to develop a better understanding, at a molecular level, of the relationships between the genetic defect and the onset of clinical features. In fact, the discovery of new genes belonging to the same pathway can lead to the introduction of new disorders that share the same cardiovascular risk as Marfan syndrome, and perhaps the same management.

Better knowledge of the underlying pathogenetic aspects can lead to personalized risk, treatment, and therapy. Eventually, increased knowledge can lead to a strong impact on the prognosis in terms of delivering a correct and early diagnosis, the correct timing of pharmacological prophylaxis, a personalized approach, personalized management, and reproductive risk calculation.

## Figures and Tables

**Table 1:** Suggested indications for follow-up in MFS.

Apparatus/System	Diagnostic Criteria and/or Related Diseases	Diagnosis and Follow-up	
		Tools	Timing
Cardiovascular	Aortic root ectasia Increased risk of aortic aneurysms and dissection Mitral Valve prolapse Increased risk of valve insufficiency	2D-transthoracic echocardiography Vascular CT/MRI thoraco-abdomen neck and CNS Cardiac Surgery evaluation	At First Evaluation, then every 6 months from the age of 18 years every 2–3 y every 3–5 y As needed
Ocular	*Ectopia Lentis* and Dislocation of the lens Myopia High risk for retinal detachment High risk for myopic based retinal degeneration Risk of glaucoma	Ophthalmological evaluation	At First evaluation, then every 12 months
Skeletal	Kyphosis/Scoliosis Dolichostenomelia Chest deformity Flat-footedness Joint laxity Protrusio Acetaboli Polyarthralgia	Orthopedic clinical evaluation Physiatric counselling and Physiotherapy Pelvis X-ray	At diagnosis, then every 12 months As needed At diagnosis
Neurovascular/Neurological	Dural ectasia CSF Hypotension	Neurological evaluation Spine MRI	at diagnosis if CSF hypotension suspected
Endocrinological	Hypovitaminosis D Osteopenia/osteoporosis	Endocrinological evaluation and Vit. D dosage BMD	every 12 months every 2 years
Respiratory	Increased risk for spontaneous pneumothorax Reduced aerobic capacity Chest deformity-linked complications	Spirometry + breathing function tests Pneumological evaluation Thoracic surgery evaluation	every 12 months As needed As needed
Integumentary and skin	Recurrent hernias Atrophic skin striae non related to weight increase, stress, or pregnancy	Surgical evaluation	As needed
Genetics	*FBN1* pathogenic variant	Counselling and genetic testing Preconceptional counselling	At First evaluation Before pregnancies
Psychological counselling and support			As Needed

## Data Availability

No new data were created or analyzed in this study.

## Supplementary Materials

The following supporting information can be downloaded at: https://www.mdpi.com/article/10.3390/diagnostics13132284/s1. Table S1: Genetically related disorders in MFS differential diagnosis due to mutations in FBN1. Table S2: Genetically related disorders in MFS differential diagnosis due to mutations in other genes.

## Abbreviations

2D-TTE: 2D-transthoracic echocardiography; ACEI: Inhibitors of Angiotensin converting enzyme; ADHD: attention-deficit/hyperactivity disorder; AGV: aortic growth velocity; AHA: American Heart Association; ARBs: Angiotensin receptor blockers; AT-1: angiotensin I; BAV: bicuspid aortic valve; BB: beta-blockers; BMD: bone mineral density; BMP: bone morphogenetic proteins; BSA: body surface area; CSF: cerebrospinal fluid; DN: dominant negative; DXA: dual energy X-ray absorptiometry; ECM: extracellular matrix; EDS: Ehlers–Danlos syndrome; EGF: epidermal growth factor; EMMPRIN: extracellular matrix metalloproteinase inducer; ESC: European Society of Cardiology; ESTVS: European Society for Thoraco-Vascular Surgery; H-TAD: Heritable Thoracic Aortic Disease; HI: haploinsufficiency mutations; MFS: Marfan syndrome; MMP: metalloproteinase; MVP: Mitral valve prolapse; LDS: Loeys–Dietz syndrome; PWV: pulse wave velocity; QoL: quality of life; TGFβ: transforming growth factor-β; TTE: Transthoracic echocardiography.
