# Supplementary material for: Marfan Syndrome: Enhanced Diagnostic Tools and Follow-up Management Strategies
Source: Diagnostics (Basel). 2023 Jul 5;13(13):2284. doi: 10.3390/diagnostics13132284 (PMC10340634; doi:10.3390/diagnostics13132284)
Supplement: Supplementary file 1 [file diagnostics-13-02284-s001.zip › diagnostics-2475178-supplementary.pdf]

Supplementary Table S1. Genetically related disorders in MFS differential diagnosis due to mutations in FBN1.

Disorder	OMIM ID	Mode of Inheritance	Gene/Protein Mutation Site
Neonatal Marfan Syndrome	No OMIM ID (ORPHA:284979)	Autosomal Dominant	- Variants between exons 23 to 32 (known as "the neonatal region")
Autosomal Dominant Weill-Marchesani Syndrome	#608328	Autosomal Dominant	- substitutions and amino acid deletion affecting domain TB5<br>- substitutions in the first hybrid domain<br>- deletion of exons 9–11 resulting in the loss of domains TB1 to EGF4
Acromicric Dysplasia	#102370	Autosomal Dominant	- Variants in exon 41 or 42, affecting domain TB5
Geleophysic dysplasia 2	#614185	Autosomal Dominant	- Variants in exon 41 or 42, affecting domain TB5
Stiff Skin Syndrome	#184900	Autosomal Dominant	- Variants affect domain TB4 which contains the only integrin-binding RGD motif of Fibrillin-1
Marfanoid Progeroid Lipodystrophy Syndrome	#616914	Autosomal Dominant	- Variants in exon 64 leading to a premature stop-codon formation in the C-terminus domain
MASS Syndrome	#604308	Autosomal Dominant	- Not defined
Ectopia lentis, familial	#129600	Autosomal Dominant	- missense variants usually involving a cysteine residue

Supplementary Table S2. Genetically related disorders in MFS differential diagnosis due to mutations in other genes.

**Disorder**	**OMIM Number**	**Gene**	**Mode of Inheritance**
Loeys-Dietz syndrome	#609192, #610168, #619656, #613795, #614816, #615582	*TGFBR1, TGFBR2, SMAD2, SMAD3, TGFB2, TGFB3*	Autosomal Dominant
Beals syndrome (Congenital contractural arachnodactyly)	#121050	*FBN2*	Autosomal Dominant
Meester-Loeys syndrome	#300989	*BGN*	X-linked
Ehlers-Danlos syndrome (EDS), classic type	#130000, #130010	*COL5A1, COL5A2*	Autosomal Dominant
EDS, Cardiac-valvular type	#225320	*COL1A2*	Autosomal Recessive
EDS, Vascular type	#130050	*COL3A1*	Autosomal Dominant
Hypermobile EDS	%130020	*Unknown*	Assumed to be Autosomal Dominant
EDS, Kyphoscoliotic form	#225400	*PLOD1, FKBP14*	Autosomal Recessive
Brittle Cornea Syndrome	#229200, #614170	*ZNF469, PRDM*	Autosomal Recessive
Arterial tortuosity syndrome	#208050	*SLC2A10*	Autosomal Recessive
Classical Homocystinuria	#236200	*CBS*	Autosomal Recessive
Stickler syndrome type I	#108300	*COL2A1*	Autosomal Dominant
Autosomal dominant polycystic kidney disease	#173900, #173910	*PKD1, PKD2*	Autosomal Dominant

Chromosome 16p13.3 duplication	#613458	*MYH11*	Autosomal Dominant (isolated cases)
Fragile X-linked syndrome	# 300624	*FMRI*	X-linked recessive
Lujan-Fryns syndrome	309520	*MED12*	X-linked recessive
Heritable Thoracic Aortic Disease	%607086	*Many genes involved*	
